# Automatic subject-specific spatiotemporal feature selection for subject-independent affective BCI

**DOI:** 10.1371/journal.pone.0253383

**Published:** 2021-08-26

**Authors:** Badar Almarri, Sanguthevar Rajasekaran, Chun-Hsi Huang

**Affiliations:** 1 Dept. of Computer Science and Engineering, University of Connecticut, Storrs, CT, United States of America; 2 Dept. of Computer Science, King Faisal University, Al-Ahsa, Saudi Arabia; 3 The School of Computing, Southern Illinois University, Carbondale, IL, United States of America; National University of Sciences and Technology, PAKISTAN

## Abstract

The dimensionality of the spatially distributed channels and the temporal resolution of electroencephalogram (EEG) based brain-computer interfaces (BCI) undermine emotion recognition models. Thus, prior to modeling such data, as the final stage of the learning pipeline, adequate preprocessing, transforming, and extracting temporal (i.e., time-series signals) and spatial (i.e., electrode channels) features are essential phases to recognize underlying human emotions. Conventionally, inter-subject variations are dealt with by avoiding the sources of variation (e.g., outliers) or turning the problem into a subject-deponent. We address this issue by preserving and learning from individual particularities in response to affective stimuli. This paper investigates and proposes a subject-independent emotion recognition framework that mitigates the subject-to-subject variability in such systems. Using an unsupervised feature selection algorithm, we reduce the feature space that is extracted from time-series signals. For the spatial features, we propose a subject-specific unsupervised learning algorithm that learns from inter-channel co-activation online. We tested this framework on real EEG benchmarks, namely DEAP, MAHNOB-HCI, and DREAMER. We train and test the selection outcomes using nested cross-validation and a support vector machine (SVM). We compared our results with the state-of-the-art subject-independent algorithms. Our results show an enhanced performance by accurately classifying human affection (i.e., based on valence and arousal) by 16%–27% compared to other studies. This work not only outperforms other subject-independent studies reported in the literature but also proposes an online analysis solution to affection recognition.

## 1 Introduction

A lack of understanding of neurophysiological signals has resulted in numerous unanswered questions about human beings, their health, and their cognitive and social development, as well as human-to-human and human-to-machine interaction. Traditionally, communication can be categorized into active (direct) or passive (indirect) communication. Active communication is contextual and can be interpreted, while passive communication involves uncertainty, and interpreting it is difficult. All means of communication (e.g., written, verbal, body language) result from neural activation in the brain. Human affection, one of the most complicated areas of investigation in cognitive and behavioral psychology, whether it occurs intrinsically (e.g., only neural activation) or extrinsically (e.g., via any ordinal method of communication), is hard to predict, communicate, and interpret. The urge to understand human brain and emotions has advanced technologies and emerging interdisciplinary fields (e.g., affective computing [1]) to recognize, interpret, and process human affects.

Emotion is a multifaceted phenomenon that can be hard to quantify for many reasons, namely, 1) individuality and event-dependence (e.g., two different people might react differently to a similar situation); 2) the existence of a broad, overlapping spectrum of emotional states; and 3) the fleeting property of emotions (i.e., emotion is momentary, as opposed to mood, which lasts longer and can be a personal trait) [2]. Two primary methods for identifying emotions: Darwinian method (i.e., universal basic emotions) and cognition (i.e., dimensional measures of valence and arousal) [3]. Cognition is a quantitative approach in which two-dimensional (i.e., arousal and valence) continuous scales represent each emotion from which other emotions can be derived. These affective measures might then correlate with neural activation in brain regions that can be detected using invasive and non-invasive techniques.

EEG is a non-invasive technique that utilizes the electrical potential from the firing of millions of neurons projected on the scalp as spatially distributed electrical potential. Compared to other non-invasive techniques, EEG has gained the interest of industry and the research community [4] thanks to 1) effective temporal resolution, measured in milliseconds; 2) reasonable cost compared to fMRI; and 3) portability and suitability for different experimental setups. However, EEG suffers from low spatial resolution and artifact interference, such as muscle movement, power frequency, and eye-blinking.

Despite the disagreement in the emotion literature regarding which regional sources are significant in identifying human emotions, it is believed that identification of positive and negative emotions occurs in the frontal, temporal, and parietal lobes. Zhang et al. *et al*. applied a channel selection procedure based on the ReliefF feature selection algorithm [5], and they concluded the electrode channels, Fp1 and T7, among other channels in the lobes mentioned above, were important. Channels Fp1 and Fp2 were frequently selected in a subject-specific sequential forward search proposed by [6]. In [7–9], the authors thoroughly reviewed and investigated different channel and feature extraction selection methods trained on several classifiers. Channel selection methods for detecting event-related potential in single-trial studies are discussed in [10]. Spatiotemporal features, however, exhibit apparent inter-subject variation, which led researchers to select a subset of subjects with less variation or prefer subject-dependent modeling instead.

In this article, we address the subject-specific particularities to recognize emotions cross-subjects. Subject-to-subject variation has been identified as one reason for low performance in subject-independent emotion recognition studies. However, subjective particularities have not been actively considered when constructing affective recognition systems. The contribution of this work includes studying subject-specific brain connectivity for individuals participating in the experiments, described in the next section, over several epochs; feature and channel (i.e., spatiotemporal features) selection using an online unsupervised learning approach; encapsulating these algorithms into the proposed affective recognition framework to recognize emotions based on features that are relevant to the participating individuals and improve the overall performance of the subject-independent affective recognition.

## 2 Materials and background

### 2.1 Datasets

DEAP is short for the Dataset of Emotional Analysis using Physiological Signals [11]. It involves 32 subjects, each of whom has watched 40 musical video clips that vary in their emotional content. The dataset consists of a multi-channel multidimensional array of size 32 * 40 * 40 * 8064 (representing subject * video * channel * data/signal). We only used 32 neurological oriented sensors. We excluded other physiological measures except for electrooculography (EOGs) for ocular artifacts removal. At the end of the session, subjects rated their affection regarding that stimulus (60 s). The frequency rate of this experiment is 512 Hz, and all data are available.

MAHNOB-HCI [12] consists of 27 subjects who watched 20 movie excerpts varying in time between (34.9–117 s) in a frequency of 512 Hz. Subjects reported their valence and arousal feedback, among other assessment reports. This experiment is similar in technical setup to DEAP, except that it does not include EOG sensors.

DREAMER dataset [13] does also test human affection through 18 music videos that vary in time (65–393 s). Twenty-three participants watched and evaluated those stimuli. The experimenter used Emotiv EPOC consisting of 14 electrode channels, recording a rate of 128 Hz.

### 2.2 Subject dependency

Emotion recognition studies can mainly be categorized into two schemes, subject-independent or subject-dependent. A subject-independent scheme is stimulus-dependent, where different neurophysiological features from different subjects contribute to the prediction outcomes based on their affective feedback. In this scheme, each stimulus is a unit, and each subject is a data point. Each participant provides feedback on their emotional states in response to stimuli in the environment. Conversely, subject-dependent learning is another scheme of affective learning implemented extensively in recent affective computing studies. Compared to the subject-dependent scheme, subject-independent emotion recognition is closer in practice to real-life applications, and it is generalizable across a population. In the subject-independent scheme, we aim to verify the validity and effect of the event from different perspectives, not only from the perspective of a single respondent. One drawback of the subject-independent scheme is that it ignores the particularities of individuals and generalizes the learning process despite the subjective differences between participants. In this work, we propose a mechanism involving personal neurological specificities to enhance the outcomes of the subject-independent scheme.

## 3 Methodology

**Fig 1:**
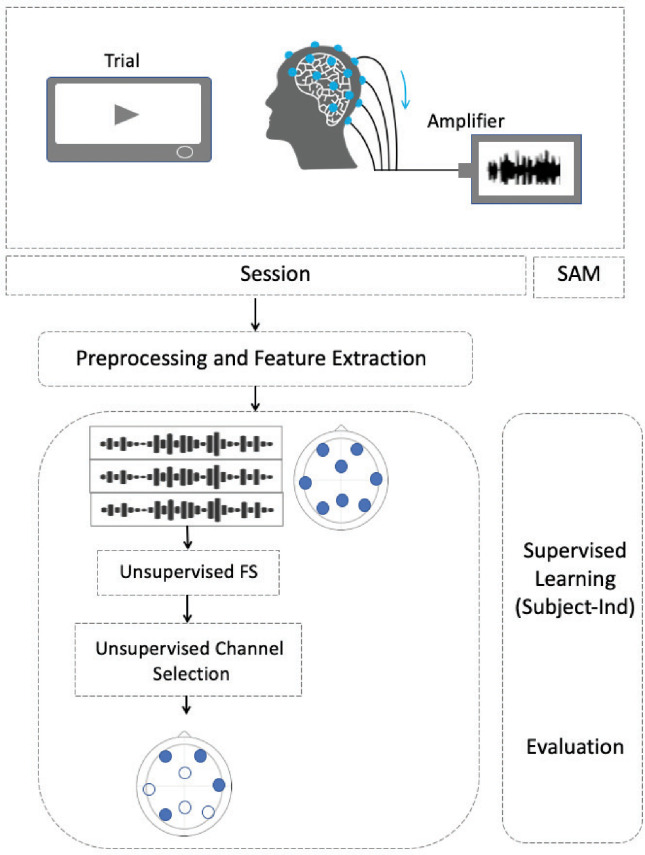
The proposed framework.

We propose an extended BCI pipeline for the automatic subject-specific unsupervised feature and channel selection for a subject-independent scheme of affect modeling and recognition. Although we mainly contributed to the channel selection, the framework was enhanced in different parts such as the unsupervised feature selection and ranking through epochs. An overview of the proposed framework is shown in Fig 1. It consists of the following phases: 1) data (i.e., EEG and EOG signals) acquisition, 2) epoching the time-series data 3) preprocessing each epoch, 4) feature extraction, 5) unsupervised channel and feature selection, 6) supervised learning, 7) evaluation and analysis.

### 3.1 Time slicing (Epoching)

Event-related sensors’ signals might be underestimated when a global analysis of the given signals is applied. Therefore, we believe dealing with smaller windows of time would lead to a higher temporal resolution; hence, feature extraction will be able to extract more relevant event-aware features [14].

We define an epoch size, referred to as *w*, and that is the synchronization point where all subjects hypothetically need to reach simultaneously for a given stimulus. *w*’s form individual units of time in which feature extraction methods are applied. We have set 3 window sizes, 2, 5, 10 seconds. Investigating the window sizes is not in the scope of this work.

### 3.2 Signal preprocessing

EEG signals are susceptible to external sources of noise (i.e., artifacts). The main external sources of EEG artifacts are power lines, eye-blinking, and muscle movement. Avoiding these undesired artifacts is almost impossible; however, post-acquisition processes might be beneficial to reduce a fair amount of these inevitable artifacts. In [9], a comprehensive survey on the EEG-based emotion studies shows that preprocessing techniques were overwhelmingly considered, which suggests the complexity, yet vitality, of this phase. In this paper, we consider the following EEG preprocessing procedure as suggested by [15, 16]:

- *Notch filter*: We removed power line noise at 50 Hz (i.e., the nominal frequency in the European standard of power) using notch filter.
- *Band-pass filter*: We applied a band-pass filter (0.5 Hz–40 Hz) corresponding to Nyquist frequency.
- *Ocular noise*:
  - To detect the eye-blinking momenta, we referenced the vertical EOG sensors (i.e. bipolar reference).
  - To exclude ocular artifacts, we applied independent component analysis, or ICA (i.e., Fast ICA [19]).
- *Down-sampling*: We down-sampled to 128 Hz to mitigate the computational and storage complexities.

This preprocessing procedure was slightly toned for each dataset due to dataset-based variations such as equipment and setups. For example, when dataset does not include ocular sources to detect eye-blinks, prefrontal electrodes can alternatively be referenced. ICA used to be a bottleneck in automating BCI applications because of the need to manual rejection of bad components. However, several algorithms to automate ICA components rejection have been developed such as [17, 18].

### 3.3 Feature extraction

After preprocessing EEG signals, we can extract features from the time-series data. Feature extraction methods can be in time-domain, frequency-domain or time-frequency. A large combination of features can be resulted from the multidimensional EEG signals and the many feature extraction algorithms presented in the EEG literature.

We use a variety of the most common feature extraction methods that have been extensively applied in BCI and emotion recognition applications [19, 20]. Below, we list the most frequently used methods in both time, frequency and time-frequency domains that we use in this work. See [19, 21] for more details. We extracted 54 features from each electrode channel (i.e. 1728 features of a 32-channel system) per subject. In parenthesis (.) are the tags we used in the figures below as a reference for the reader.

- Time-domain
  - Moments: Mean (mean), Variance (var), Standard Deviation (std), Skewness (skw), Kurtosis (kurts).
  - Entropy: Approximate Entropy (entrpA), Sample Entropy (entrpS), Spectral Entropy (entrpSp).
  - Hjorth: Hjorth mobility and complexity (hjorthM, hjorthC).
  - Others: Peak to peak (ptp), Higuchi Fractal Dimension (higuchi), Katz Fractal Dimension (katz), Number of zero-crossings (zcross), Line length (lnlen), Decorrelation time (decorr), Hurst exponent of the data (hurst).
- Frequency-domain
  - Spectral Edge Frequency (spec_frq), Hjorth mobility and complexity of power spectrum (hjorthMsp, hjorthCsp), loglog scale of the linear regression of power spectral density, PSD (spc_slp1-spc_slp4)^⋆^, Band energy (enrg_bnd1-enrg_bnd5)^†^, Power Spectrum (powfreq1-powfreq5)^†^.
    ^⋆^ 1-4 = Spectral slope’s intercept, slope, MSE, and R2; ^†^ 1-5 = Frequency bands: delta (0.5–4 Hz), theta (4–8 Hz), alpha (8–12 Hz), beta (12–30 Hz), and gamma (30–Nyquist frequency Hz).
- Time-frequency-domain
  - discrete wavelet transform (DWT) coefficients (wvlet1-wvlet6)^◇^, Teager-Kaiser energy (tk1-tk14)^◇^.
    ^◇^ 1-6 = levels of decomposition DWT; ^◇^ 1-14 = (DWT levels of decomposition+1)*2

### 3.4 Unsupervised feature extraction selection

Feature selection is a dimensional reduction process that aims to represent data in a low-dimensional space by selecting a subset of its original feature space. It approaches this goal by eliminating irrelevant and redundant features.

Based on where a feature selection method is designed to occur in the learning pipeline, feature selection can be generally categorized into four categories, filter, wrapper, embedded, and hybrid methods [9, 22]. The latter two methods are derived from either filter or wrapper or a combination of both. Filter methods employ the selection criteria regardless to the outcomes of the classification algorithm, thus reducing the effect of over-fitting. In contrast, wrapper methods is highly dependent on the process of learning where the outcome of the learner decides whether or not certain features are to be eliminated. Unlike filter methods, wrapper methods involve extensive computation since the majority of the learning pipeline recursively search for an optimized selection of features.

This framework is to select features in an online unsupervised manner. For this purpose, several unsupervised algorithms such as Laplacian score and dispersion ratio have been reported in many reported studies for feature selection. Here, we use Laplacian score feature selection to rank the time and frequency features for a subject-specific episodic signals generated from different sources (i.e. channels). Laplacian score algorithm works as follows:

- Construct a k-nearest neighbor graph G.
- Define a similarity matrix *S* populated as (1):
  $$\begin{array}{c} S_{ij}=\{\begin{array}{cc} e^{-\frac{{\left\|x_{i}-x_{j}\right\|}^{2}}{t}}, & i,j\in G^{k}\\ 0, & otherwise \end{array} \end{array}$$ (1)
- Compute the graph Laplacian for each feature *r* using L = D − S, where D is the diagonal matrix.
- Finally, a Laplacian score is calculated for each feature *f*_*r*_,
  $$\begin{array}{c} L_{r}=\frac{\sum_{ij}{\left(f_{ri}-f_{rj}\right)}^{2}S_{ij}}{Var(f_{r})} \end{array}$$ (2)
  and the goal is to minimize the objective function. Features with lower scores are ranked higher.

### 3.5 Unsupervised subject-specific channel selection algorithm

Channel selection can contextually be seen as a sub-domain of feature selection. EEG signals are multi-dimensional where a number of channels detect and record simultaneous signals from different regions of the brain. Temporal signals are then transformed to more informational features.

We present a novel algorithm that aims to find a representative subset of the channel space by learning from inter-channel interactivity while subjects are still in session. Basing this work on unsupervised similarity-based learning, channel selection known as a time-consuming task can now be online. The ultimate goals to this phase is to minimize unnecessary redundancy in the channel space and reduce computational and storage overheads.

#### 3.5.1 Inter-channel connectivity learning

Here, we propose an inter-channel connectivity-based learning algorithm that learns subject-specific connectivity patterns over epochs.

**Notation**: In this section, let $X_{\hat{T}}^{C*F}$ be a subject-trial-independent data encapsulated in a tensor form as $X_{\hat{T}}^{C*F}=\left\{X_{\hat{t}}^{c*f}:\hat{t}=1,\ldots ,\hat{T}\in N,c=1,\ldots ,C\in N,f=1,\ldots ,F\in N\right\}$ where $\hat{t}$ refers to epochs (i.e. time-windows) of a given time-series, and *c* ∈ *C* is a set of electrode channels, each of which has *f* ∈ *F* feature vector. A similarity matrix $S\in R^{n*n}$ is the pairwise similarity among instances of a given dimension. For instance, a pairwise similarity among channels is calculated along the electrode channel dimension ∀*c* ∈ *C*. Pairwise relations are best described as graphs since similarity among these instances can be encoded in an affinity graph. We define a graph *G* = (*V*, *E*) where *V* is a set of vertices or nodes (i.e. electrode channels) and *E* is the edge-set of *G*. *G* is an undirected symmetric graph where an edge *e*_*uv*_ is the similarity measure (i.e. correlation) between two nodes (i.e. electrode channels) 〈*u*, *v*〉.

**Fig 2:**
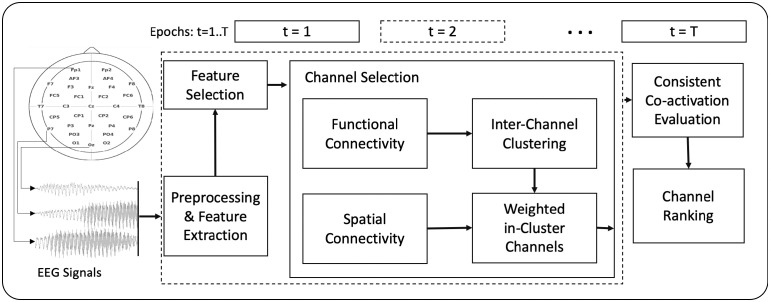
A general diagram of the proposed channel selection algorithm. The dotted box corresponds to a specific window of time (e.g., t = 2).

**Algorithm**: Given $X_{\hat{T}}^{C*F}$, we aim to find a subset of electrode channels $\hat{C}\leq C$ without adversely compromising the representability compared to when we use the whole channel space *C*. In the following, we provide a narrative explanation of the algorithm. An illustrative diagram is shown in Fig 2.

Prior to investigating inter-channel functional connectivity, we first construct a neighboring matrix that encodes EEG electrode channels’ spatial distribution. The neighboring matrix is denoted as *δ* ∈ *R*^c*c^, and each entry is populated by a distance measure between every two channels in the channel space, as given by (3).

$$\begin{array}{c} \delta \left(p,q\right)=\{\begin{array}{cc} \kappa , & p\neq q\;\&\;\kappa \leq \kappa_{max}\\ 0, & otherwise \end{array} \end{array}$$ (3)

In (3), *δ* is a topological distance function that calculates the shortest path of how far node *p* is from node *q*. *κ* is the unweighted edge distance between any two nodes of the function *δ* such that it does not exceed a preset maximum value *κ*_*max*_.

Independent of the spatial distribution, we assess how similar (i.e., correlated) electrode channels among each other are in respect to functional neural co-activation among different electrode channels. For that, we measure inter-channel similarities given their feature spaces. Using an efficient similarity measure is greatly vital. Euclidean distance, correlation, and mutual information are reportedly used on such data [23]. Here, we use Pearson’s correlation (4).

$$\begin{array}{c} S\left(c_{i},c_{j}\right)=\frac{COV(X{}_{t}^{c_{i}*f},X{}_{t}^{c_{j}*f})}{\sqrt{Var\left(X{}_{t}^{c_{i}*f}\right)Var\left(X{}_{t}^{c_{i}*f}\right)}} \end{array}$$ (4)

where *i* and *j* are any two electrode channels *c* ∈ *R*^*f*^, where *f* is the feature space, and *i* ≠ *j*.

Now we cluster electrode channels that synchronously co-activate based on how similar inter-channel attributes are in a given window of time. Electrode channels assigned to different clusters are less similar in terms of their time and frequency attributes. The time-variant channel synchrony increases the complexity of detecting co-activation between different regions of the brain. Therefore, a clustering method that discovers co-activations in all regions without extra presumptions such as a predefined number of clusters or a high dependency on random initialization is favorable. Hence, we apply affinity propagation that iteratively yields data samples to exchange messages in order to conclude, upon convergence, either a data sample is a cluster exemplar or one associated with another data sample identified as an exemplar-based on a similarity matrix *S*_*ij*_. In order to identify exemplars and cluster other data points accordingly, AP defines two matrices [24]: responsibility *ρ* and availability *α* as shown in Eqs (5) and (6), respectively.

$$\begin{array}{c} \rho_{ik}=S_{ik}-\underset{k^{\prime }\neq k}{\text{max}}\left(S_{ik^{\prime }}+\alpha_{ik^{\prime }}\right) \end{array}$$ (5)

$$\begin{array}{c} \alpha_{ik}=\{\begin{array}{cc} \text{min}[0,\rho_{kk}+\sum_{j\notin \{i,k\}}\text{max}(0,\rho_{jk})], & i\neq k\\ \sum_{j\neq k}\text{max}(0,\rho_{jk}), & i=k \end{array} \end{array}$$ (6)

where i and k are any data sample and potential exemplar, respectively. Eq (6) updates the on- and off-diagonal of *α*. Ultimately, each data sample will be assigned to an exemplar that seems most similar to it and also selected by other samples. Exemplars and their associated data samples form the final clusters. The final co-activation functional clusters resulted from the set of exemplars *K* in {*α*_*kk*_ + *ρ*_*kk*_ > 0} and the assignment process ∀*i* ≠ *k* ∈ *n* are depicted as labeling matrix, L as shown in (7).

$$\begin{array}{c} L_{ij}=\{\begin{array}{cc} 1, & i,j\in k\in K\;\&\;i\neq j\\ 0, & otherwise \end{array} \end{array}$$ (7)

With the knowledge of the functional clusters, presented in Eq (7), as well as spatial distribution of channels, we construct a graph *G* that represents concurrent activation among nodes (i.e. electrode channels). Spatially close electrode channels often read similar signals due to projection of underlying neurons, so we assign lesser weights on their corresponding edges according to their spatial closeness, Eq (3).

This algorithm select different sets of channels in subsequent epochs. Hence, to decide what subject-specific channels should be eventually involved, we evaluate outcomes of subsequent epochs and limit the selection to epochs that exhibit consistency in the way channels co-activate. This reduces the random electric potential that is caused by remaining artifacts and/or non-event related neural activities. Finally, we rank electrode channels based on their consistency through epochs as well as their functional over spatial correlation.

### 3.6 Best-k subject-independent channel and feature selection via mutual information estimation

In an unsupervised fashion, we designed the above pipeline to extract features and select channels and their associated features based on individuals’ reception of stimuli presented in the three experiments. The proposed channel selection and the feature selection algorithms rank subjective channels and features based on neurological connectivity across epochs of a stimulus. It is practical to infer the importance of channels and features in real-time to achieve online emotion recognition. Since labeling is not available until the end of an experiment, we count on unsupervised learning to infer the underlying features and channel importance. Once subjective labeling is available, deciding top k channels and features is more feasible. A subset of inter-subject channel space and its extracted features are then selected to train the affect recognition model.

Estimating mutual information (MIE) between individual features and the target variable depicts how two random variables (*x*, *y*) are relevant. Given a multivariate data *X* ∈ *R*^*x*^ where *x* represents a feature vector and a target variable *y*, we estimate *MI*(*x*, *y*) for each feature vector as:

$$\begin{array}{c} MI(x,y)=H(x)+H(y)-H(x,y) \end{array}$$ (8)

where *H*(.) is the marginal entropy and *H*(., .) is the joint entropy; both can be derived to:

$$\begin{array}{c} MI\left(x,y\right)=\sum_{x,y}p\left(x,y\right)\log\frac{p(x,y)}{p(x)p(y)} \end{array}$$ (9)

where *p*(.) is the probability distribution function.

Features that are strictly irrelevant to the target variable will weigh zero, hence, eliminated. The higher the mutual information estimation value is, the more relevant the feature is to the target.

### 3.7 Learning and cross-validation

To classify emotions, we use a support vector machine (SVM) to make comparisons with other works fair and to show the merit of the proposed framework. Using grid search, the regularizing parameter C is set in (.025,.5,1,10,100) for both linear and radial basis function (RBF) kernels (RBF’s kernel scale = [0.001, 2, 10,50,100]). Since it is not the focus of this work, we do not intensively parametrize or emphasize the parametric outcomes of these kernels.

We used nested cross-validation to eliminate data leakage from the MI-based feature selection to the testing set. The inner cross-validation is for mutual information estimation to select the inter-subject temporal and spatial features ranked in a previous step based on individual subjects. That is an all-but-one training set and a novel subject assigned into the testing set of the outer cross-validation. MIE or minimum redundancy maximum relevance (mRMR) are usually used to search through all combinations of channels and features exhaustively in previous works. Here, since we minimize the spatial and temporal feature spaces earlier in this framework, MIE is now left with a smaller set of features to measure how relevant they are to the target emotion.

## 4 Results and discussion

Here, we present the performance of our proposed framework applied to 3 real data benchmarks, namely DEAP, MAHNOB-HCI, and DREAMER. The early phases of the pipeline are epoching, preprocessing, and feature extraction. We used different feature extraction methods in the time, frequency, and time-frequency domains. Then we applied feature and channel selection algorithms to extract the most reliable and relevant features and their sources (i.e., electrode channels). We used Laplacian scoring for temporal features and a proposed algorithm for a subject-specific channel selection. We encapsulated these algorithms in the subject-independent emotion recognition framework, where we learn features in an unsupervised manner until target variables (i.e., subjective labeling) are available. We estimated the relevance of ranked features to two target emotions, valence and arousal, and built a nested cross-validated model for emotion recognition.

### 4.1 Temporal and spectral feature selection

**Fig 3:**
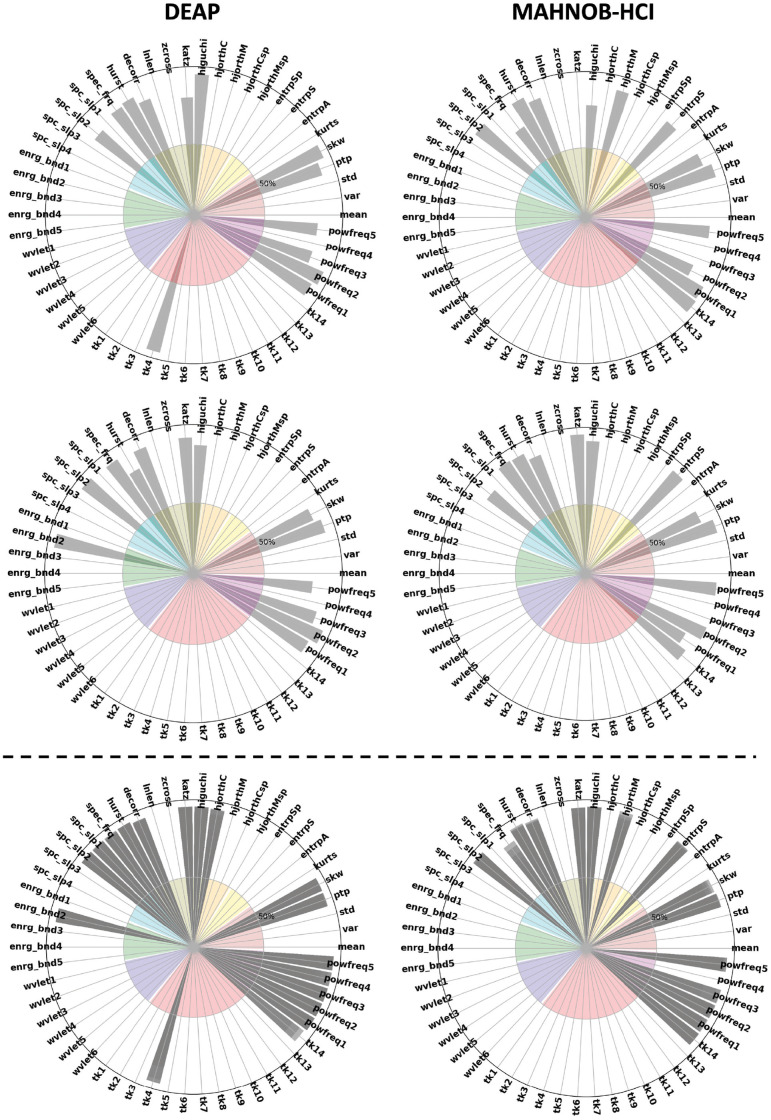
Frequently selected features based on the proposed framework in DEAP and MAHNOB-HCI (DREAMER’s was excluded for better resolution). The colors of the inner circle represent groups of feature extraction methods (statistical, entropy, Hjorth, power spectrum band and energy, Wavelet coefficients and Teager-Kaiser energy). Columns represent how many times a specific feature was selected in an experiment. Below the dashed line is the feature selection frequency across stimuli (i.e. intensity of grey indicates that feature is highly favorable).

Although there was some variation in features selected using the Laplacian score, mutual information estimation of features against the subjective labels minimized this variation across subjects. Fig 3 shows features selected more frequently in each stimulus. Only a third of the pool of features were selected in the benchmarks we used in this work.

**Fig 4:**
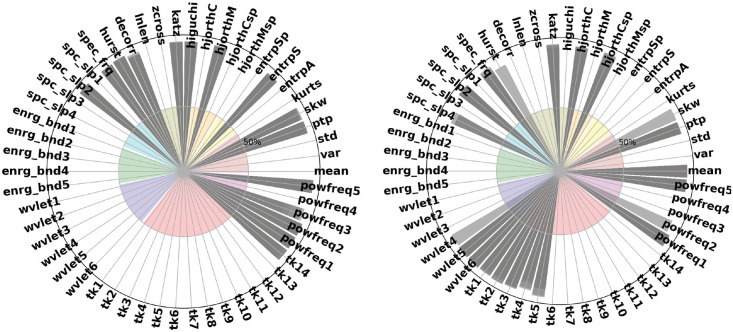
The effect of epoch (window) size on feature selection. We compare window sizes of w = 10s (left) and 2s (right).

Features that explain the spectral properties of the power spectrum like the average power of theta, alpha, and beta are of great importance to recognize emotions, see Figs 3 and 4. Additionally, temporal features that infer information directly from the signal in the time-domain were selected, such as statistical moments (i.e., mean and skewness), Hjorth parameters, and methods that measure regulatory and wave unpredictability in time-series (e.g., entropy).

Similar features have been reported in the literature on emotion recognition. The average power of the frequency bands theta, alpha, and beta, and the mean and skewness of the time-series signal were selected in [25] using Fisher Criterion Score (FCS). Authors of [22] reported their analysis of selecting features based on Chi-square, mutual information, ANOVA F-value, and recursive feature elimination (RFE). They showed that beta and gamma bands are more correlated to the dependent emotion variable. Most of the recent literature on channel and feature selection relied heavily on supervised techniques that would not advance online emotion recognition. Temporal and spatial feature selection algorithms we used and proposed in this work are independent of the subjective feedback except for measuring the relevance between the few ranked channels and features with the target emotion.

### 4.2 Channel selection

**Fig 5:**
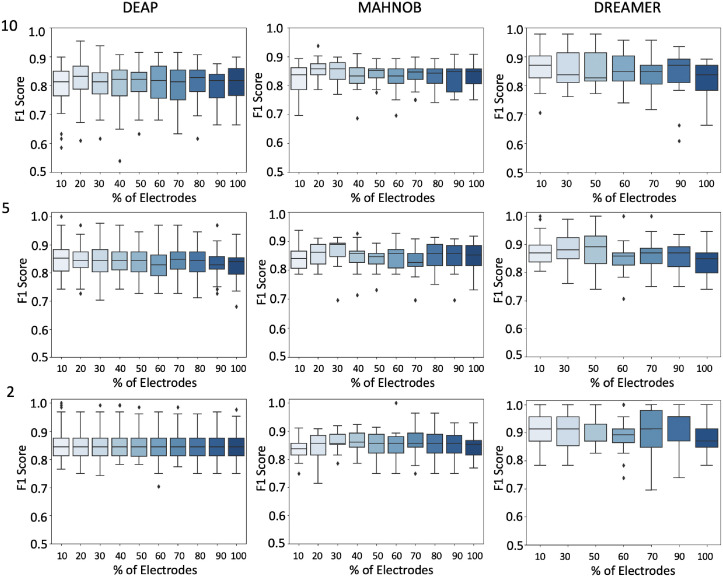
Valence: F1 scores for various ranked electrode channels selections (10%,20%,…,100%) on the x-axis, and F1 score on the y-axis. We show datasets vertically, and epoch sizes (10s, 5s, and 2s) horizontally. Each box plot depicts f1 scores across trials.

**Fig 6:**
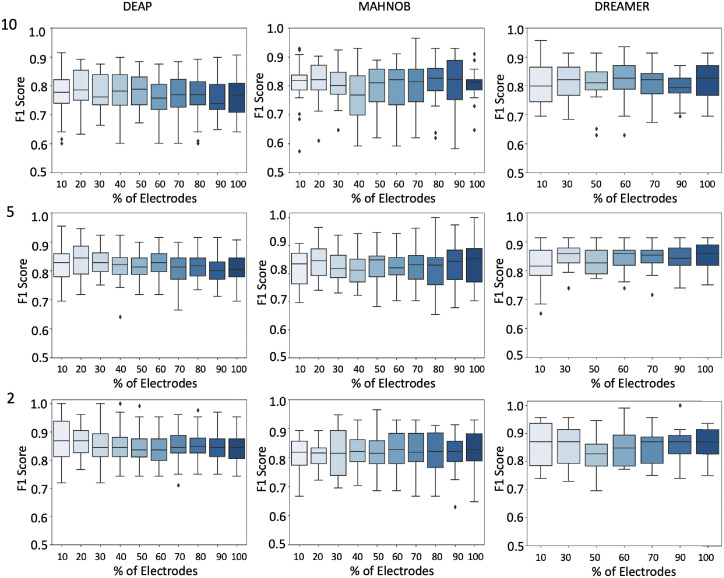
Arousal: F1 scores for various ranked electrode channels selections (10%,20%,…,100%) on the x-axis, and F1 score on the y-axis. We show datasets vertically, and epoch sizes (10s, 5s, and 2s) horizontally. Each box plot depicts f1 scores across trials.

We used the channel selection algorithm proposed in this work to rank electrode channels to reduce redundancy and exhibit consistent regional co-activation during the stimulus session. In Figs 5 and 6, we can see that a number of channels between 4-10 are sufficiently competitive based on the results we got here in this study and compared to other studies. We noticed the smaller the epoch size is, the more accurate we can classify emotions. This can be justified by the detailed coverage of features when extracted based on smaller windows of time.

**Fig 7:**
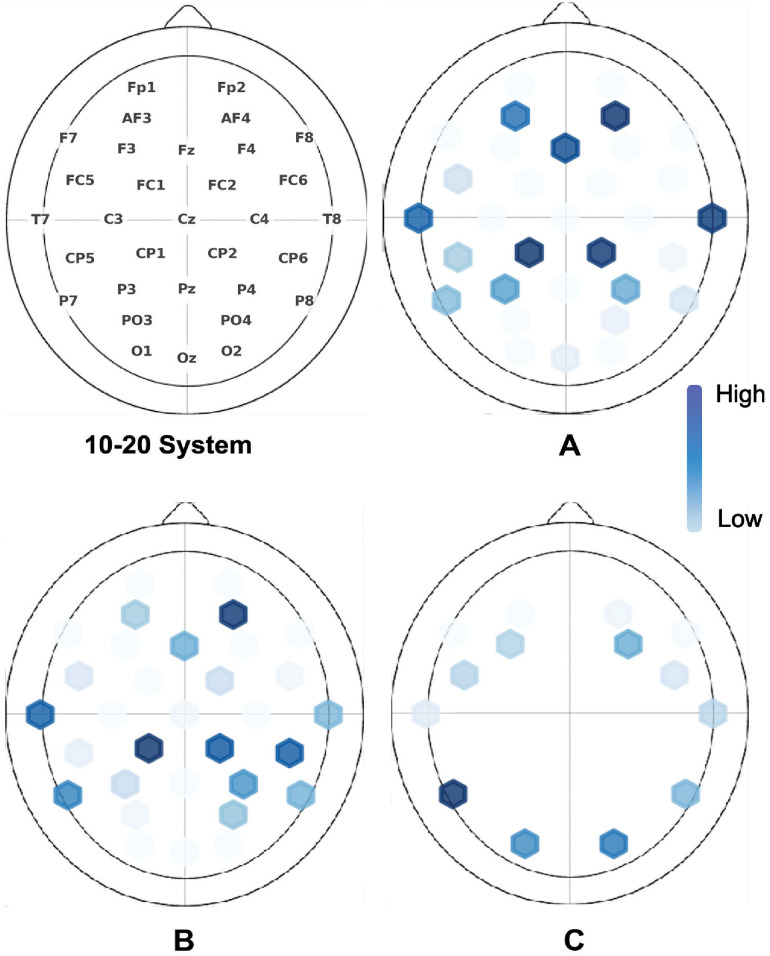
Cross-session channel selection of A) DEAP, B) MAHNOB-HCI, and C) DREAMER. Color intensity represent the frequency of selecting a channel. Top-left is the 10-20 system for EEG electrode placement system.

According to the emotion literature, the brain’s frontal lobe mainly projects human emotions through neural activation. Most of the studies on public datasets included frontal lobe electrodes such as F3 and F4. Using the feature selection methods mentioned in the previous section, electrode channels and their temporal signals are usually reduced with less consideration to the spatial distribution of channels. Electrode channels and their temporal signals are conventionally transformed to a feature vector before a feature selection is applied. Here, we address the spatial distribution of the multidimensional feature space. We show that our unsupervised results align with the literature in DEAP, MAHNOB, and DREAMER datasets, see Fig 7. Frontal, temporal, and occipital lobes are frequently selected as shown in Fig 7.

### 4.3 Performance comparison

We compare our results with works that studied emotion recognition using a subject-independent scheme, which is considerably more challenging than subject-dependent.

We used a SVM classifier to predict the emotional state of a novel subject given their EEG data. We used dual cross-validation and grid search to optimize the SVM kernels and their parameters mentioned in 3.7. We found that the regularization parameter of one (i.e., C = 1) generally gives higher accuracy across datasets. Linear SVM are more stable; hence, the results are based on a linear SVM.

Our results outperformed the existing subject-independent emotion recognition by an improvement of about 16%–27%. We achieved a performance in classifying human affection in an online fashion in different datasets as follows: DEAP (0.88, 0.86), MAHNOB-HCI (0.87, 0.82), and DREAMER (0.89, 0.85) for (valence and arousal). Performance was measured using F1 score due to the imbalanced classes. All studies we compare with but [26] reported their results as aggregated values (e.g., grand mean); hence, detailed statistical analyses for all studies are hard to conduct. We used a one-sided Wilcoxon signed-rank test to test our results against that of [26] given their leave-one-subject-out results of DEAP. It validated that our results are comparatively statistically significant (p <.05) based on the valence results of all stimuli mean of 68 ± 34% compared to that reported here of 88 ± 7%. We show our results compared to the recently published works in Table 1.

**Table 1:** Comparison between our study and similar studies in the emotion literature.

	Year	Eval Metric	Classifier	Score	
				Valence	Arousal
DEAP Dataset					
Chen J. et al. [25]	2017	Acc	SVM	0.78*	-
		F1		-	-
Soheil R. et al. [27]	2018	Acc	RF	0.59	0.56
		F1		0.50	0.57
Xiang Li et al. [28]	2018	Acc	SVM	0.59	-
		F1		-	-
Pallavi P. et al. [29]	2019	Acc	DNN	0.63	0.64
		F1		-	-
Miguel A-H. et al. [30]	2019	Acc	SVM	0.58	0.56
		F1		-	-
Yucel C. et al. [26]	2020	Acc	SVM	0.73	-
		F1		-	-
Our Work	2020	Acc	SVM	0.88	0.86
		F1		0.88	0.86
MAHNOB Dataset					
Haiyan X. et al. [31]	2015	Acc	SVM	0.63	0.65
		F1		-	-
Ferdinando H. et al. [32]	2018	Acc	kNN	0.75	0.78
		F1		-	-
Our Work	2020	Acc	SVM	0.87	0.82
		F1		0.87	0.82
DREAMER Dataset					
Miguel A-H. et al. [30]	2019	Acc	SVM	0.59	0.58
		F1		-	-
Hector G. et al. [33]	2019	Acc	CNN	0.70	0.72
		F1		-	-
Our Work	2020	Acc	SVM	0.89	0.85
		F1		0.89	0.85

* combined target (Valence/Arousal)

## 5 Conclusion

We propose a subject-independent framework to recognize human affects by learning individual particularities in an unsupervised manner. That is, the most stimulus-subject-relevant EEG features and channels are selected to identify regions of the brain that activate due to a given stimulus. We then applied mutual information estimation to select features that are more relevant to the target emotion to level out inter-subject variation. Using nested cross-validation and an SVM classifier, we tested the proposed framework on real EEG data, namely DEAP, MAHNOB-HCI, and DREAMER. We were able to classify human affection in real-time as accurate as 0.88, 0.87, and 0.89 for valence; and 0.86, 0.82, and 0.85 for arousal in DEAP, MAHNOB-HCI DREAMER, respectively. This framework also proposes an online analysis solution for affect recognition. Our work achieves a performance increase of 16–27% for valence and arousal compared to the recently published results of emotion recognition using the subject-independent scheme, which is considerably more challenging than subject-dependent schemes.

## References

[1] Picard RW. Affective computing. MIT press; 2000.

[2] Mulligan K, Scherer KR. Toward a working definition of emotion. Emotion Review. 2012;4(4):345–357. doi: 10.1177/1754073912445818

[3] Bos DO, et al. EEG-based emotion recognition. The Influence of Visual and Auditory Stimuli. 2006;56(3):1–17.

[4] Rojas GM, Alvarez C, Montoya CE, de la Iglesia-Vayá M, Cisternas JE, Gálvez M. Study of resting-state functional connectivity networks using EEG electrodes position as seed. Frontiers in neuroscience. 2018;12:235. doi: 10.3389/fnins.2018.00235 29740268 PMC5928390

[5] Zhang J, Chen M, Zhao S, Hu S, Shi Z, Cao Y. ReliefF-based EEG sensor selection methods for emotion recognition. Sensors. 2016;16(10):1558. doi: 10.3390/s16101558 27669247 PMC5087347

[6] Aydemir O, Ergün E. A robust and subject-specific sequential forward search method for effective channel selection in brain computer interfaces. Journal of neuroscience methods. 2019;313:60–67. doi: 10.1016/j.jneumeth.2018.12.004 30529410

[7] Al-Nafjan A, Hosny M, Al-Ohali Y, Al-Wabil A. Review and classification of emotion recognition based on EEG brain-computer interface system research: a systematic review. Applied Sciences. 2017;7(12):1239. doi: 10.3390/app7121239

[8] Alotaiby T, Abd El-Samie FE, Alshebeili SA, Ahmad I. A review of channel selection algorithms for EEG signal processing. EURASIP Journal on Advances in Signal Processing. 2015;2015(1):66. doi: 10.1186/s13634-015-0251-9

[9] Jenke R, Peer A, Buss M. Feature extraction and selection for emotion recognition from EEG. IEEE Transactions on Affective computing. 2014;5(3):327–339. doi: 10.1109/TAFFC.2014.2339834

[10] Feess D, Krell MM, Metzen JH. Comparison of sensor selection mechanisms for an ERP-based brain-computer interface. PloS one. 2013;8(7):e67543. doi: 10.1371/journal.pone.0067543 23844021 PMC3699630

[11] Koelstra S, Muhl C, Soleymani M, Lee JS, Yazdani A, Ebrahimi T, et al. Deap: A database for emotion analysis; using physiological signals. IEEE transactions on affective computing. 2011;3(1):18–31. doi: 10.1109/T-AFFC.2011.15

[12] Soleymani M, Lichtenauer J, Pun T, Pantic M. A multimodal database for affect recognition and implicit tagging. IEEE transactions on affective computing. 2011;3(1):42–55. doi: 10.1109/T-AFFC.2011.25

[13] Katsigiannis S, Ramzan N. DREAMER: A database for emotion recognition through EEG and ECG signals from wireless low-cost off-the-shelf devices. IEEE journal of biomedical and health informatics. 2017;22(1):98–107. doi: 10.1109/JBHI.2017.2688239 28368836

[14] Ehrlich SK, Agres KR, Guan C, Cheng G. A closed-loop, music-based brain-computer interface for emotion mediation. PloS one. 2019;14(3):e0213516. doi: 10.1371/journal.pone.0213516 30883569 PMC6422328

[15] Islam MK, Rastegarnia A, Yang Z. Methods for artifact detection and removal from scalp EEG: A review. Neurophysiologie Clinique/Clinical Neurophysiology. 2016;46(4-5):287–305. 27751622 10.1016/j.neucli.2016.07.002

[16] Brunner C, Billinger M, Vidaurre C, Neuper C. A comparison of univariate, vector, bilinear autoregressive, and band power features for brain–computer interfaces. Medical & biological engineering & computing. 2011;49(11):1337–1346. 21947797 10.1007/s11517-011-0828-x PMC3208819

[17] Nolan H, Whelan R, Reilly RB. FASTER: fully automated statistical thresholding for EEG artifact rejection. Journal of neuroscience methods. 2010;192(1):152–162. doi: 10.1016/j.jneumeth.2010.07.015 20654646

[18] Mognon A, Jovicich J, Bruzzone L, Buiatti M. ADJUST: An automatic EEG artifact detector based on the joint use of spatial and temporal features. Psychophysiology. 2011;48(2):229–240. doi: 10.1111/j.1469-8986.2010.01061.x 20636297

[19] Wagh KP, Vasanth K. Electroencephalograph (EEG) based emotion recognition system: A review. Innovations in Electronics and Communication Engineering. 2019; p. 37–59. doi: 10.1007/978-981-10-8204-7_5

[20] Boonyakitanont P, Lek-Uthai A, Chomtho K, Songsiri J. A review of feature extraction and performance evaluation in epileptic seizure detection using EEG. Biomedical Signal Processing and Control. 2020;57:101702. doi: 10.1016/j.bspc.2019.101702

[21] Badani S, Saha S, Kumar A, Chatterjee S, Bose R. Detection of epilepsy based on discrete wavelet transform and Teager-Kaiser energy operator. In: 2017 IEEE Calcutta Conference (CALCON). IEEE; 2017. p. 164–167.

[22] Li J, Cheng K, Wang S, Morstatter F, Trevino RP, Tang J, et al. Feature selection: A data perspective. ACM Computing Surveys (CSUR). 2017;50(6):1–45. doi: 10.1145/3136625

[23] Aghabozorgi S, Shirkhorshidi AS, Wah TY. Time-series clustering–a decade review. Information Systems. 2015;53:16–38. doi: 10.1016/j.is.2015.04.007

[24] Frey BJ, Dueck D. Clustering by passing messages between data points. science. 2007;315(5814):972–976. doi: 10.1126/science.1136800 17218491

[25] Chen J, Hu B, Wang Y, Moore P, Dai Y, Feng L, et al. Subject-independent emotion recognition based on physiological signals: a three-stage decision method. BMC medical informatics and decision making. 2017;17(3):167. doi: 10.1186/s12911-017-0562-x 29297324 PMC5751758

[26] Cimtay Y, Ekmekcioglu E. Investigating the use of pretrained convolutional neural network on cross-subject and cross-dataset EEG emotion recognition. Sensors. 2020;20(7):2034. doi: 10.3390/s20072034 32260445 PMC7181114

[27] Rayatdoost S, Soleymani M. Cross-corpus EEG-based emotion recognition. In: 2018 IEEE 28th International Workshop on Machine Learning for Signal Processing (MLSP). IEEE; 2018. p. 1–6.

[28] Li X, Song D, Zhang P, Zhang Y, Hou Y, Hu B. Exploring EEG features in cross-subject emotion recognition. Frontiers in neuroscience. 2018;12:162. doi: 10.3389/fnins.2018.00162 29615853 PMC5867345

[29] Pandey P, Seeja K. Subject independent emotion recognition from EEG using VMD and deep learning. Journal of King Saud University-Computer and Information Sciences. 2019;. doi: 10.1016/j.jksuci.2019.11.003

[30] Arevalillo-Herráez M, Cobos M, Roger S, García-Pineda M. Combining Inter-Subject Modeling with a Subject-Based Data Transformation to Improve Affect Recognition from EEG Signals. Sensors. 2019;19(13):2999. doi: 10.3390/s19132999 31288378 PMC6651152

[31] Xu H, Plataniotis KN. Subject independent affective states classification using EEG signals. In: 2015 IEEE Global Conference on Signal and Information Processing (GlobalSIP). IEEE; 2015. p. 1312–1316.

[32] Ferdinando H, Alasaarela E. Emotion recognition using cvxEDA-based features. Journal of Telecommunication, Electronic and Computer Engineering (JTEC). 2018;10(2-3):19–23.

[33] Gonzalez HA, Yoo J, Elfadel IAM. EEG-based Emotion Detection Using Unsupervised Transfer Learning. In: 2019 41st Annual International Conference of the IEEE Engineering in Medicine and Biology Society (EMBC). IEEE; 2019. p. 694–697. 10.1109/EMBC.2019.8857248 31945992

